# Supplementary material for: Comprehensive Analysis of the PP2C Gene Family in Grape (Vitis vinifera L.) and Identification of VvPP2C26 and VvPP2C41 as Negative Regulators of Fruit Ripening
Source: Plants (Basel). 2025 Dec 16;14(24):3827. doi: 10.3390/plants14243827 (PMC12736828; doi:10.3390/plants14243827)
Supplement: Supplementary file 1 [file plants-14-03827-s001.zip › plants-4013473-supplementary.pdf]

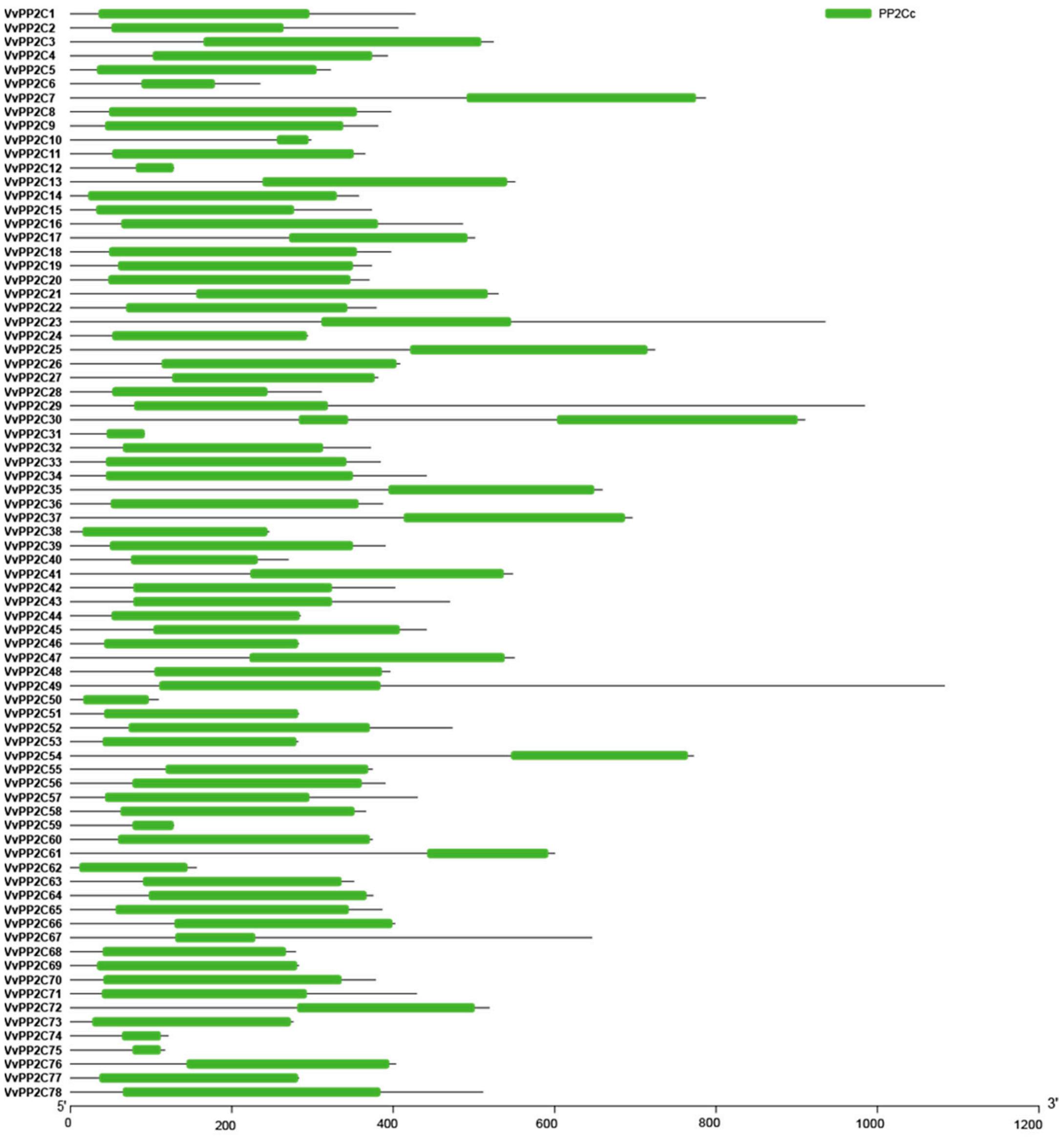

Figure S1. Conserved domains analysis of VvPP2Cs.

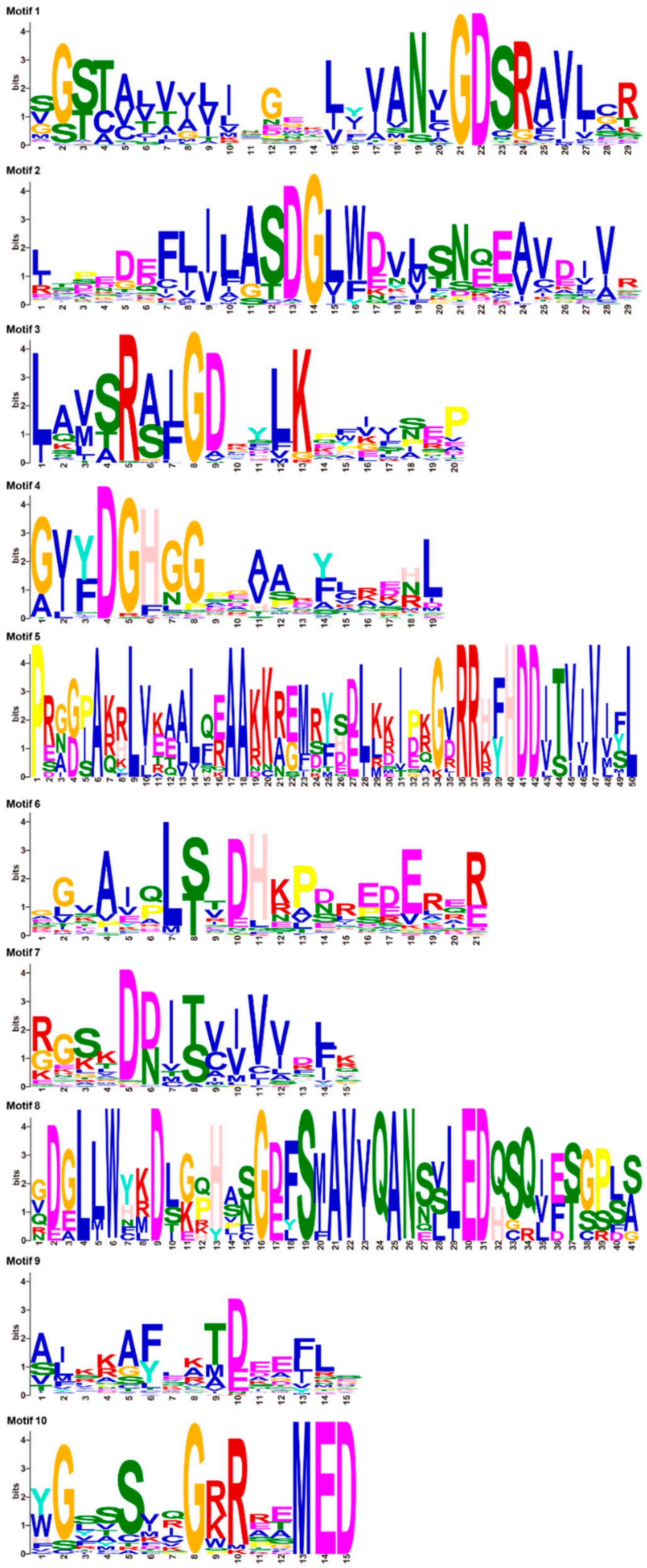

Figure S2. Sequence logos of 10 conserved motifs detected in the VvPP2C proteins. The overall height of each stack represents the degree of conservation at this position, while the height of the individual letters within each stack indicates the relative frequency of the corresponding amino acid.

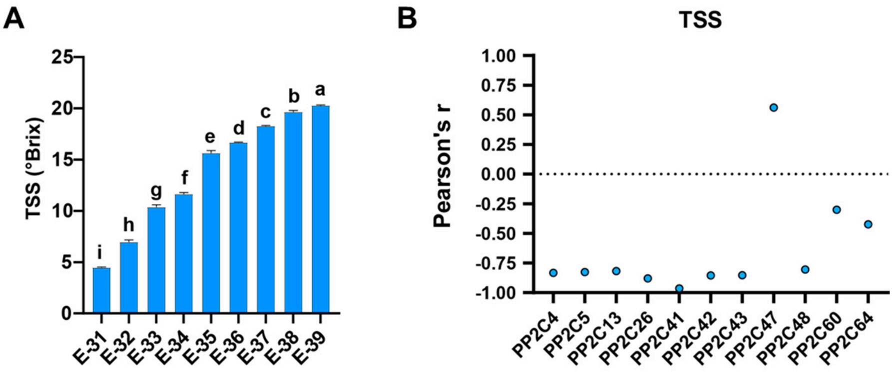

Figure S3. Correlation between *VvPP2CA* expression and soluble solids during berry development. (A) Total soluble solids (TSS) content in ‘Kyoho’ grape berries at different developmental stages. Error bars represent SD (n=3). Different lowercase letters indicate significant differences ($P < 0.05$, Duncan’ s multiple range test). (B) Pearson correlation analysis of VvPP2CA gene expression with TSS content.

Table S1. The duplication pairs of *VvPP2C* genes in grapevine

Segmental duplication pairs	
VvPP2C47	VvPP2C13
VvPP2C48	VvPP2C42
VvPP2C47	VvPP2C41
VvPP2C51	VvPP2C77
VvPP2C55	VvPP2C27
VvPP2C56	VvPP2C36
VvPP2C64	VvPP2C4
VvPP2C70	VvPP2C9
VvPP2C72	VvPP2C17
VvPP2C69	VvPP2C32
VvPP2C70	VvPP2C33
VvPP2C8	VvPP2C18
VvPP2C9	VvPP2C33
VvPP2C25	VvPP2C37

Tandem duplication pairs	
VvPP2C4	VvPP2C5
VvPP2C6	VvPP2C7
VvPP2C11	VvPP2C12
VvPP2C12	VvPP2C13
VvPP2C27	VvPP2C28
VvPP2C42	VvPP2C43
VvPP2C48	VvPP2C49
VvPP2C57	VvPP2C58
VvPP2C61	VvPP2C62
VvPP2C63	VvPP2C64

Table S2. The duplication pairs of VvPP2C genes in grape and Arabidopsis thaliana.

Segmental duplication pairs	
VvPP2C1	PAC:19657198
VvPP2C7	PAC:19639831
VvPP2C8	PAC:19646061
VvPP2C9	PAC:19647669
VvPP2C18	PAC:19659727
VvPP2C16	PAC:19664142
VvPP2C11	PAC:19647070
VvPP2C14	PAC:19646473
VvPP2C18	PAC:19646061
VvPP2C15	PAC:19647357
VvPP2C17	PAC:19672498
VvPP2C11	PAC:19672326
VvPP2C18	PAC:19665514
VvPP2C15	PAC:19666100
VvPP2C24	PAC:19662575

VvPP2C19	PAC:19660634
VvPP2C20	PAC:19664884
VvPP2C19	PAC:19659231
VvPP2C22	PAC:19660264
VvPP2C23	PAC:19665618
VvPP2C27	PAC:19655597
VvPP2C26	PAC:19654645
VvPP2C25	PAC:19654204
VvPP2C25	PAC:19640485
VvPP2C26	PAC:19641764
VvPP2C27	PAC:19638502
VvPP2C28	PAC:19639099
VvPP2C27	PAC:19642233
VvPP2C26	PAC:19671063
VvPP2C25	PAC:19667262
VvPP2C31	PAC:19653874
VvPP2C30	PAC:19641962
VvPP2C29	PAC:19663472
VvPP2C37	PAC:19654204
VvPP2C35	PAC:19641737
VvPP2C37	PAC:19640485
VvPP2C37	PAC:19659685
VvPP2C36	PAC:19660187
VvPP2C34	PAC:19667875
VvPP2C36	PAC:19670669
VvPP2C37	PAC:19667262
VvPP2C39	PAC:19669156
VvPP2C41	PAC:19657804

VvPP2C41	PAC:19656972
VvPP2C47	PAC:19657804
VvPP2C49	PAC:19640167
VvPP2C47	PAC:19647923
VvPP2C47	PAC:19669918
VvPP2C52	PAC:19649097
VvPP2C51	PAC:19642509
VvPP2C51	PAC:19647084
VvPP2C52	PAC:19646077
VvPP2C55	PAC:19642233
VvPP2C55	PAC:19638502
VvPP2C56	PAC:19660187
VvPP2C56	PAC:19670669
VvPP2C57	PAC:19656844
VvPP2C58	PAC:19659231
VvPP2C58	PAC:19667986
VvPP2C65	PAC:19643909
VvPP2C60	PAC:19673327
VvPP2C63	PAC:19668272
VvPP2C66	PAC:19649910
VvPP2C67	PAC:19669143
VvPP2C69	PAC:19653874
VvPP2C69	PAC:19650566
VvPP2C69	PAC:19655099
VvPP2C71	PAC:19654899
VvPP2C73	PAC:19654123
VvPP2C72	PAC:19672498
VvPP2C77	PAC:19664079

Table S3. The duplication pairs of *VvPP2C* genes in grape and tomato

Segmental duplication pairs	
VvPP2C1	Solyc05g009070.3.1
VvPP2C5	Solyc03g006940.2.1
VvPP2C4	Solyc03g007230.3.1
VvPP2C4	Solyc08g082260.2.1
VvPP2C7	Solyc08g077150.3.1
VvPP2C7	Solyc08g007000.3.1
VvPP2C8	Solyc01g111730.3.1
VvPP2C9	Solyc01g107300.3.1
VvPP2C8	Solyc10g055650.2.1
VvPP2C9	Solyc10g049630.2.1
VvPP2C8	Solyc02g083420.3.1
VvPP2C9	Solyc02g092750.3.1

VvPP2C18	Solyc01g111730.3.1
VvPP2C18	Solyc10g055650.2.1
VvPP2C18	Solyc02g083420.3.1
VvPP2C18	Solyc03g033340.3.1
VvPP2C17	Solyc04g080090.3.1
VvPP2C16	Solyc04g056560.3.1
VvPP2C16	Solyc05g055790.3.1
VvPP2C13	Solyc07g040990.3.1
VvPP2C11	Solyc08g065670.3.1
VvPP2C13	Solyc08g062650.3.1
VvPP2C19	Solyc01g100040.3.1
VvPP2C19	Solyc01g065700.3.1
VvPP2C23	Solyc01g079710.3.1

VvPP2C22	Solyc01g080400.3.1
VvPP2C21	Solyc01g066870.3.1
VvPP2C24	Solyc09g090280.2.1
VvPP2C25	Solyc10g086490.2.1
VvPP2C25	Solyc06g076100.3.1
VvPP2C26	Solyc06g076400.3.1
VvPP2C27	Solyc06g082080.3.1
VvPP2C27	Solyc06g007200.3.1
VvPP2C28	Solyc06g007350.3.1
VvPP2C25	Solyc09g010780.3.1
VvPP2C32	Solyc01g105280.3.1
VvPP2C33	Solyc02g092750.3.1
VvPP2C32	Solyc04g074180.3.1
VvPP2C29	Solyc06g084100.3.1
VvPP2C29	Solyc09g065650.3.1
VvPP2C39	Solyc10g078820.2.1
VvPP2C37	Solyc10g086490.2.1
VvPP2C34	Solyc10g085370.2.1
VvPP2C34	Solyc10g076320.2.1
VvPP2C36	Solyc10g084410.2.1
VvPP2C37	Solyc06g076100.3.1
VvPP2C37	Solyc09g010780.3.1
VvPP2C36	Solyc09g007080.3.1
VvPP2C41	Solyc03g121880.3.1
VvPP2C41	Solyc07g040990.3.1
VvPP2C41	Solyc08g062650.3.1
VvPP2C47	Solyc12g096020.2.1
VvPP2C47	Solyc03g121880.3.1

VvPP2C49	Solyc05g018300.3.1
VvPP2C47	Solyc07g040990.3.1
VvPP2C47	Solyc08g062650.3.1
VvPP2C52	Solyc10g008490.3.1
VvPP2C53	Solyc04g074180.3.1
VvPP2C51	Solyc07g053760.3.1
VvPP2C51	Solyc07g062970.3.1
VvPP2C52	Solyc07g054300.3.1
VvPP2C55	Solyc05g052520.3.1
VvPP2C55	Solyc06g082080.3.1
VvPP2C56	Solyc09g007080.3.1
VvPP2C58	Solyc01g100040.3.1
VvPP2C57	Solyc01g100110.3.1
VvPP2C65	Solyc03g082970.3.1
VvPP2C63	Solyc03g007270.3.1
VvPP2C60	Solyc03g006920.3.1
VvPP2C66	Solyc03g118890.3.1
VvPP2C69	Solyc01g105280.3.1
VvPP2C70	Solyc10g049630.2.1
VvPP2C72	Solyc12g089090.2.1
VvPP2C70	Solyc02g092750.3.1
VvPP2C71	Solyc04g079120.3.1
VvPP2C72	Solyc04g080090.3.1
VvPP2C73	Solyc04g064500.3.1
VvPP2C78	Solyc10g005640.3.1
VvPP2C77	Solyc07g062970.3.1
VvPP2C78	Solyc07g066260.3.1
VvPP2C77	Solyc07g053760.3.1

Table S4. The numbers and genome size of *PP2C* homologous genes found in indicated species

Specie	Number of PP2C	Genome Size (Mb)
Arabidopsis thaliana	80	135
Cucumis sativus	56	242
Fragaria vesca	56	215
Fragaria ananassa	228	793
Arachis hypogaea	178	2607
Triticum aestivum	257	14600
Glycine max	134	1014
Vitis vinifera	78	500

Table S5. Sequence of primers used for expression analysis, F for the former primer, R for the rear primer.

Experimen ts	Primers	Sequence (5'→3')
**qRT-PCR**		
VvPP2C4	*VvPP2C4*-qPCR-F	ACTGGCAATGGCTCAAGGAA
	VvPP2C4-qPCR-R	ATGGCTTGGGCTGTTTGGAT
VvPP2C5	*VvPP2C5*-qPCR-F	ACCGGCTGCACAAGTTAGTT
	VvPP2C5-qPCR-R	TCGCTGGCTTCGACGTTAAT
VvPP2C13	*VvPP2C13*-qPCR-F	TCTGCTTTGGCCGAGGAAAT
	VvPP2C13-qPCR-R	AACCAACGGTTTCTGGAGCA
VvPP2C26	*VvPP2C26*-qPCR-F	AAGTTCGGCATAGCATCGGT
	VvPP2C26-qPCR-R	TCTGCAGTTCATCGCCACAT
VvPP2C41	*VvPP2C41*-qPCR-F	AGCGGTGACATGATTCAGCA
	VvPP2C41-qPCR-R	CCAGCAACCAAAGCCAACAA
VvPP2C42	*VvPP2C42*-qPCR-F	AGTCTCCTTTGCTGCACTGA
	VvPP2C42-qPCR-R	AAGCCTGCCAATCTAGACCT
VvPP2C43	*VvPP2C43*-qPCR-F	TGGCCTATTTCTGCGTTACC
	VvPP2C43-qPCR-R	ATGATCCTCGTAGCAGTCCT

VvPP2C47	*VvPP2C47*-qPCR-F	AGAACAGTTAGCGACAGCGT
	VvPP2C47-qPCR-R	GAAATTGGGCACAGCTGCAA
VvPP2C48	*VvPP2C48*-qPCR-F	TGGCTCTCAGGTGGCTAAAT
	VvPP2C48-qPCR-R	ACATTGTCGGCCCTCTCAAA
VvPP2C60	*VvPP2C60*-qPCR-F	ACAGAGTCGTCGTTGTCCAT
	VvPP2C60-qPCR-R	TTTGCACGGATACCGCATCT
VvPP2C64	*VvPP2C64*-qPCR-F	TGCATGTCAAGTTGTGAGGA
	VvPP2C64-qPCR-R	AAGATTGCTTTCCCTCGTCC
**Expression vector**		
VvPP2C26-pBWA(V)HS	*VvPP2C26*-*Eco31I*-pBWA(V)HS-F	GGAGAGAACACGGGGGACTTTGCAACGGTCTCATGGCGGAGATCTGCTGTG
	VvPP2C26-*Eco31I*-pBWA(V)HS-R	CAGTACTGAAGACAGAGCTAGTTACAGGTCTCCTACGTGTCCTTCCTAAGATCC
VvPP2C41-pBWA(V)HS	*VvPP2C41*-*Eco31I*-pBWA(V)HS-F	GGAGAGAACACGGGGGACTTTGCAACGGTCTCATGGAAGAGATGTCTCCTGCAG
	VvPP2C4-*Eco31I*-pBWA(V)HS-R	CAGTACTGAAGACAGAGCTAGTTACAGGTCTCTTATGAGGGTTTACTCTTGAACTTCC
